# A public, cross-reactive glycoprotein epitope confounds Ebola virus serology

**DOI:** 10.1002/jmv.29946

**Published:** 2024-10

**Authors:** Markus H. Kainulainen, Jessica R. Harmon, Elif Karaaslan, Jackson Kyondo, Amy Whitesell, Sam Twongyeirwe, Jason H. Malenfant, Jimmy Baluku, Aaron Kofman, Éric Bergeron, Michelle A. Waltenburg, Luke Nyakarahuka, Stephen Balinandi, Caitlin M. Cossaboom, Mary J. Choi, Trevor R. Shoemaker, Joel M. Montgomery, Christina F. Spiropoulou

**Affiliations:** 1 Viral Special Pathogens Branch, Division of High-Consequence Pathogens and Pathology, Centers for Disease Control and Prevention, Atlanta, Georgia, USA; 2 VHF Diagnostics Laboratory, Department of Arbovirology, Emerging and Re-emerging Infectious Diseases, Uganda Virus Research Institute, Entebbe, Uganda; 3 Department of Biosecurity, Ecosystems, and Veterinary Public Health, College of Veterinary Medicine, Animal Resources, and Biosecurity, Makerere University, Kampala, Uganda

**Keywords:** Ebola virus, epitope mapping, false positive reactions, glycoprotein, immunodominant site, serologic tests

## Abstract

Ebola disease (EBOD) in humans is a severe disease caused by at least four related viruses in the genus *Orthoebolavirus*, most often by the eponymous Ebola virus. Due to human-to-human transmission and incomplete success in treating cases despite promising therapeutic development, EBOD is a high priority in public health research. Yet despite almost 50 years since EBOD was first described, the sources of these viruses remain undefined and much remains to be understood about the disease epidemiology and virus emergence and spread. One important approach to improve our understanding is detection of antibodies that can reveal past human infections. However, serosurveys routinely describe seroprevalences that imply infection rates much higher than those clinically observed. Proposed hypotheses to explain this difference include existence of common but less pathogenic strains or relatives of these viruses, misidentification of EBOD as something else, and a higher proportion of subclinical infections than currently appreciated. The work presented here maps B-cell epitopes in the spike protein of Ebola virus and describes a single epitope that is cross-reactive with an antigen seemingly unrelated to orthoebolaviruses. Antibodies against this epitope appear to explain most of the unexpected reactivity towards the spike, arguing against common but unidentified infections in the population. Importantly, antibodies of cross-reactive donors from within and outside the known EBOD geographic range bound the same epitope. In light of this finding, it is plausible that epitope mapping enables broadly applicable specificity improvements in the field of serology.

## 1 | INTRODUCTION

Several orthoebolaviruses (family *Filoviridae*) are known to cause severe, often lethal disease in humans. Outbreaks of Ebola disease (EBOD) resulting from Ebola (EBOV), Sudan (SUDV), Bundibugyo (BDBV) and Taï Forest (TAFV) virus infections occur in parts of Sub-Saharan Africa.^1^ Typically, these outbreaks begin when a virus spreads from an unidentified host to a human, with person-to-person transmission frequently ensuing. However, in the aftermath of large epidemics caused by EBOV in the past decade, new outbreaks sparked by transmission from individuals persistently infected during previous outbreaks appear to have become more common.^2^ The source of transmission is an important distinction from the public health standpoint, since ecological work to understand and mitigate zoonotic spillover and survivor programs to identify and support convalescent individuals are both needed to prevent EBOD emergence. Historically, EBOV is the most common orthoebolavirus to cause human outbreaks and two vaccines, both based on viral vectors, have been approved to prevent disease caused by it.^3–6^

Diagnosis of acute EBOD relies primarily on reverse transcription-polymerase chain reaction- (RT-PCR) based assays, the sensitivity and specificity of which are typically higher than those of current rapid antigen tests.^7,8^ Outside acute case diagnostics, serological testing for antibodies binding viral proteins is important for various purposes. Contacts of known cases can be screened to understand transmission and frequency of asymptomatic cases. Inhabitants of potentially affected areas are surveyed to understand how often the disease goes unnoticed or misdiagnosed. Antibodies against the viral glycoprotein are quantified to study the approved EBOV vaccines. These goals set different requirements for assay performance, since false positive results confound conclusions more when true positivity is lower. The best-case scenario for control purposes is arguably a prospective vaccine study. Seroconversion is detected and individuals seropositive at baseline can be taken into consideration at the analysis stage. On the other hand, serological surveys of EBOD case contacts can be improved by carefully selecting a local negative control group to account for other possible sources of reactivity, whatever they may be.^9,10^ Perhaps most challenging is the quantification of unidentified cases in the general population, because demarcating a meaningful control group from the same location is difficult. Indeed, use of multiple methods and substantial variation between results have yielded no consensus despite a number of serosurveys published^11,12^ and meta-analysis of 26 EBOV seroprevalence studies in apparently healthy individuals found 8% seroprevalence.^13^ Finding such common evidence of past infection is difficult to reconcile with the high mortality seen in verified cases.^13^ This has led to discussion regarding potential causes of this high seropositivity, including outbreak misidentification, higher than appreciated proportion of subclinical infections, and the presence of more common but less virulent relatives of known orthoebolaviruses.^14^

In the present work, we study unexplained antibody reactivity against the EBOV attachment and fusion glycoprotein GP_1,2_, which is the primary viral antigen in the approved EBOV vaccines^3–6^ and an increasingly used recombinant antigen in serology tests. Antibodies binding EBOV GP_1,2_ are regularly found at frequencies of 2%–4% or higher even in Europe and Northern America, which lie outside the known geographic range of orthoebolavirus emergence.^15–19^ The results point to a single cross-reactive epitope that is recognized by strikingly similar antibodies in donors from both Africa and Northern America and that can be disabled by specific residue changes without compromising antigen production yield. This suggests that the primary cause of the frequent EBOV GP_1,2_ antibody finding is neither infection by an unknown orthoebolavirus nor subclinical or misidentified EBOV infection and proposes ways for broad improvements in the field of serological assays.

## 2 | METHODS

### 2.1 | Peptides

Peptides with N-terminal biotin-AHX modification were synthesized commercially (GenScript). All peptides except for screen peptides P17, P23, P34, P35, P47, and P57 were delivered at purities equal to or exceeding 70%. The peptides were dissolved in H_2_O, 10%–25% acetic acid, 50–125 mM ammonium bicarbonate, or DMSO, depending on solubility, to yield 1 mg/ml stocks, which were stored at −80°C.

### 2.2 | Protein expression and purification

The trimeric ectodomain of EBOV GP_1,2_ (isolate H.sapiens-tc/COD/1995/Kikwit-9510621) was expressed and purified as previously described.^20^ Briefly, a codon-optimized sequence encoding GP_1,2_ residues 1–647 (as listed in Uniprot P87666) with stabilizing residue changesT577P and K588F^21^ and fused to C-terminal His and AviTags was cloned into an expression vector. Expi293F cells (Thermo Fisher) were transiently transfected using FectoPro transfection reagent (Polyplus) and supernatants harvested 4 days later by removing cells via centrifugation and by removing debris via 0.2-micron polyethersulfone membrane filtration. The protein product was purified on HisTrap Excel columns (Cytiva), the AviTag was specifically biotinylated with BirA biotin-protein ligase (Avidity), and the biotinylated protein was purified by size exclusion chromatography on a Superdex 200 pg column (Cytiva). The resulting antigens were aliquoted in PBS and stored at −80°C. EBOV GP_1,2_ trimers with specific residue changes in the identified epitope (as described in the main text) were produced by the same workflow.

### 2.3 | ELISA

**FIGURE 1:**
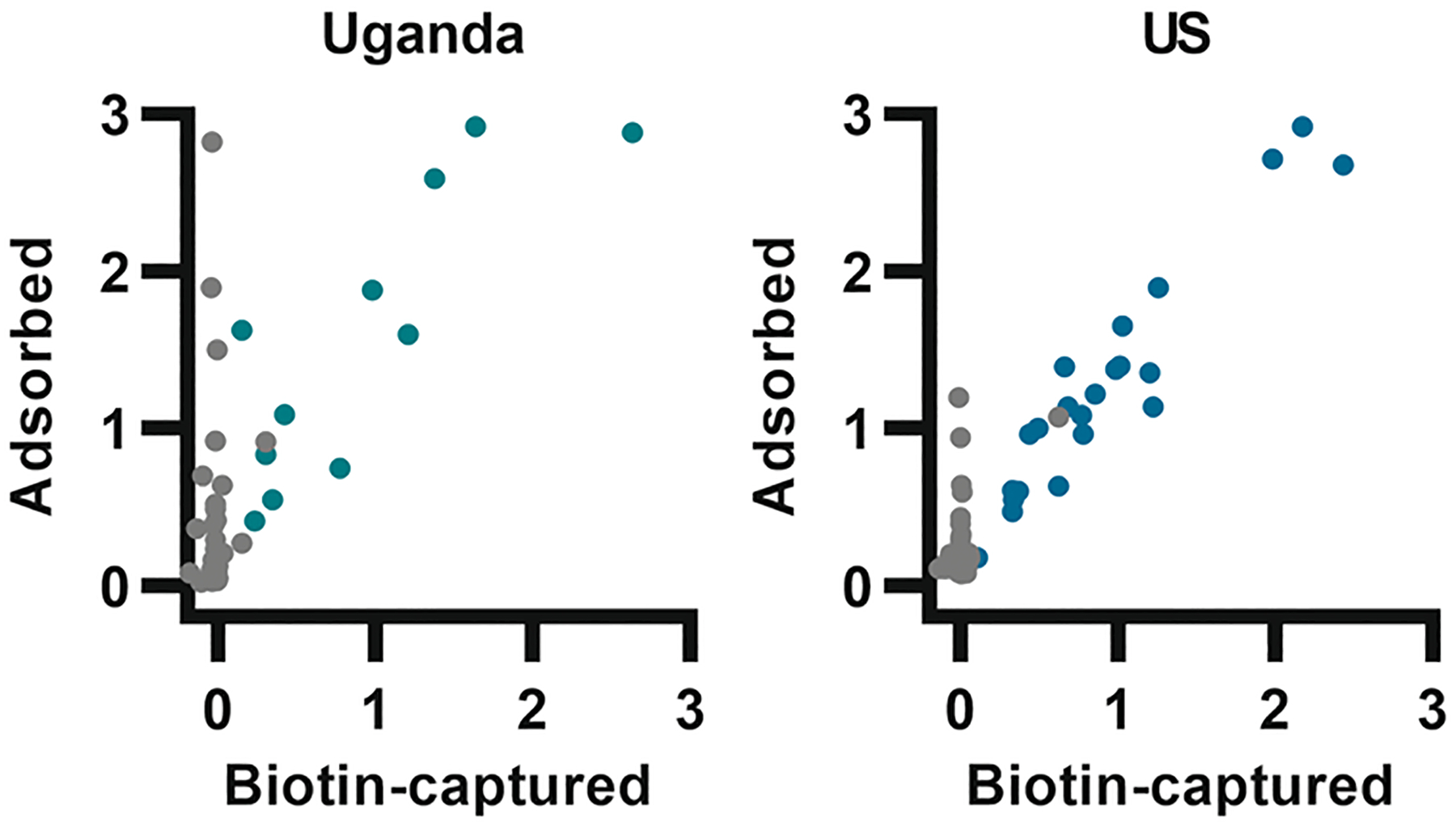
Frequent unspecific binding of IgG antibodies to adsorbed but not biotin-captured EBOV GP_1,2_ antigen. A trimeric EBOV GP_1,2_ antigen was either adsorbed to assay surface or captured via StrepTactin-biotin interaction. Left: serum samples from vaccinated individuals from Uganda (green; *N* = 11) were compared to serum samples from unvaccinated individuals from Uganda (gray; *N* = 54). Right: vaccinated individuals (teal; *N* = 22) and unvaccinated individuals (gray; *N* = 102) from the US. The numbers represent optical density values.

For biotin capture of EBOV GP_1,2_ trimers and peptides, 25 ng Step-Tactin/well (IBA Lifesciences) in PBS was coated onto MaxiSorp 384-well plates (Nunc) at +4°C overnight. The plates were washed three times with 0.1% Tween-20 (Sigma-Aldrich) in PBS and blocked using 5% milk powder (Cell Signaling Technology) in the same at room temperature (RT). After the blocking solution was discarded, 12.5 ng biotinylated peptide or GP_1,2_ trimer was added in the blocking buffer and captured for 1 h at RT. After washes, serum samples (heated for 30 min at +56°C) were added at indicated dilutions for 1 h at RT. After washing, bound IgG molecules were detected by anti-human IgG-HRP (Jackson ImmunoResearch) diluted 1:10,000 in blocking buffer, followed by washes and signal development with 1-Step Ultra TMB substrate (Thermo Fisher), followed by ELISA Stop Solution (Thermo Fisher) 10 min later. Resulting 450 nm absorbance signals (Synergy Neo2 instrument; BioTek) were corrected by subtracting values from no-sample wells and sample-specific no-antigen wells. For adsorption format, GP_1,2_ antigens were coated at 12.5 ng/well of a 384-well plate overnight at +4°C. The above protocol was then followed, except that second blocking step was done instead of antigen capture when the two formats were run in parallel. In the experiments shown in Figures 1 and S2, MaxiSorp 96-well plates were coated with 50 ng Strep-Tactin followed by 50 ng biotinylated GP_1,2_ trimer, or Immulon 2HB plates were coated directly with 50 ng antigen trimers. All experiments used technical duplicates.

### 2.4 | Peptide scan

ELISA was conducted as noted above with technical duplicates. Cut-off for positive signal in the peptide scan was set at 0.25 background-corrected absorbance units. The median signal of the 64 peptides across 81 individuals was −0.0015 and the mean signal was 0.0376, with no normal distribution observed. A few donor serum samples showed weak reactivity against continuous stretches of peptides, which was considered unlikely to represent true reactivity against specific epitopes and the chosen cut-off renders these signals negative. With this cut-off, 3.1% of the donor/peptide pairs were considered positive. For the purposes of the peptide scan, the same cut-off was applied to the trimeric GP_1,2_ antigen. Each sample was tested once in technical duplicates and data from several experiments are presented in a single figure.

### 2.5 | IgG depletions

To remove antibodies binding specific peptides from serum, Dyna-beads M-270 streptavidin beads (Thermo Fisher) were first coupled to biotinylated peptides at 5 μg peptide/mg beads in PBS for 1 h at RT. After washing with PBS, the beads were blocked in 5% nonfat dry milk and 0.1% Tween-20 in PBS for 1 h at RT. Heat-treated serum samples (17.5 μl at final dilution 1:200) were then incubated with 0.125 mg beads in blocking buffer for 1 h at RT. The resulting depleted product was collected by removing the beads using a magnet.

### 2.6 | Biolayer interferometry

Biolayer interferometry measurements were done in 0.5% bovine serum albumin and 0.05% Tween-20 in PBS using an Octet R8 instrument (Sartorius) run at +30°C, shaking at 1000 rpm. Biotinylated wild-type and variant EBOV GP_1,2_ trimers were bound to streptavidin sensors at 5 μg/ml. Baseline, mAb association at 5 μg/ml, and mAb dissociation were then measured in the same buffer.

### 2.7 | Antibodies

The monoclonal antibodies KZ52, c13C6 FR1, and c6D8 were obtained from IBT Bioservices (products 0260–001, 0201–023, and 0201–021, respectively). The monoclonal antibodies ADI-15878^22^ and mAb114,^23^ as well as the negative control mAb, were expressed in Expi293F cells and purified by Protein A affinity chromatography.

### 2.8 | Samples

The samples used in this study were collected under IRB-approved protocols (US vaccinees; CDC, Uganda donors^24^; CDC and Uganda Ministry of Health, Ebola survivors^25^; CDC and Liberia Ministry of Health). The serum samples were collected >1 year from vaccination or clinical disease. US healthy donor serum samples for negative control purposes were obtained from a commercial source and the donors were reported by the provider to be negative for hepatitis B virus surface antigen and nucleic acid, HIV-1 and HIV-2 antibodies and nucleic acids, hepatitis C virus antibody and nucleic acid, syphilis, West Nile virus nucleic acid, and at least on one previous donation negative for antibodies against Trypanosoma cruzi.

### 2.9 | Statistics

Statistical analyses were done using GraphPad Prism software (version 9.0.0). Fisher’s exact test with two-sided p-values was used to assess association of reactivity with sex and vaccination status. Two-tailed Mann-Whitney test was used to compare age distributions of donors that provided reactive and nonreactive samples. One-sample Wilcoxon signed-rank test was used to compare the signal difference from wild-type and variant antigens against no-difference hypothesis (signal difference 0). Signal correlations from different assays were determined using Spearman correlation with two-tailed p-values. For all tests, significance was determined at p-value threshold of 0.05.

## 3 | RESULTS

EBOV structural glycoprotein GP_1,2_ is expressed as a single protein that is subsequently cleaved into disulfide-linked subunits GP_1_ (containing core, glycan cap and mucin-like domains) and GP_2_ (anchoring to viral membrane and containing the fusion domain). With the help of two stabilizing residue changes and removal of the transmembrane region, the protein can be expressed as a soluble trimer.^21^ During establishment of an assay for detecting IgG antibodies against EBOV GP_1,2_ in vaccinated healthcare workers from Uganda,^24^ adsorbing the antigen to the assay surface, which is the standard procedure in such assays, frequently caused high background signal from unvaccinated individuals. However, when the same antigen was bound via biotin specifically coupled to an area corresponding to the deleted trans-membrane region in the native protein, no such background signal was detected (Figure 1). Similar results were obtained when studying vaccinated and unvaccinated individuals from the United States (US). The observation suggests that adsorption of the GP_1,2_ antigen via nonspecific interactions exposed epitopes not accessible by serum antibodies when the antigen was specifically captured via the C-terminus, and that those interactions could make unvaccinated individuals appear seropositive. This reactivity among unvaccinated donors was not statistically correlated with sex (7/85 of males and 5/71 of females reactive at one standard deviation over mean, Fisher’s exact test *p* > 0.99) or age (median of seven Uganda reactive donors: 27 years, range 23–60 vs. 47 nonreactive: 27 years, 18–60, and five US reactive donors: 32 years, range 28–45 vs. 97 nonreactive: 41, 18–71, Mann-Whitney test *p* = 0.48 and 0.14, respectively.)

**FIGURE 2:**
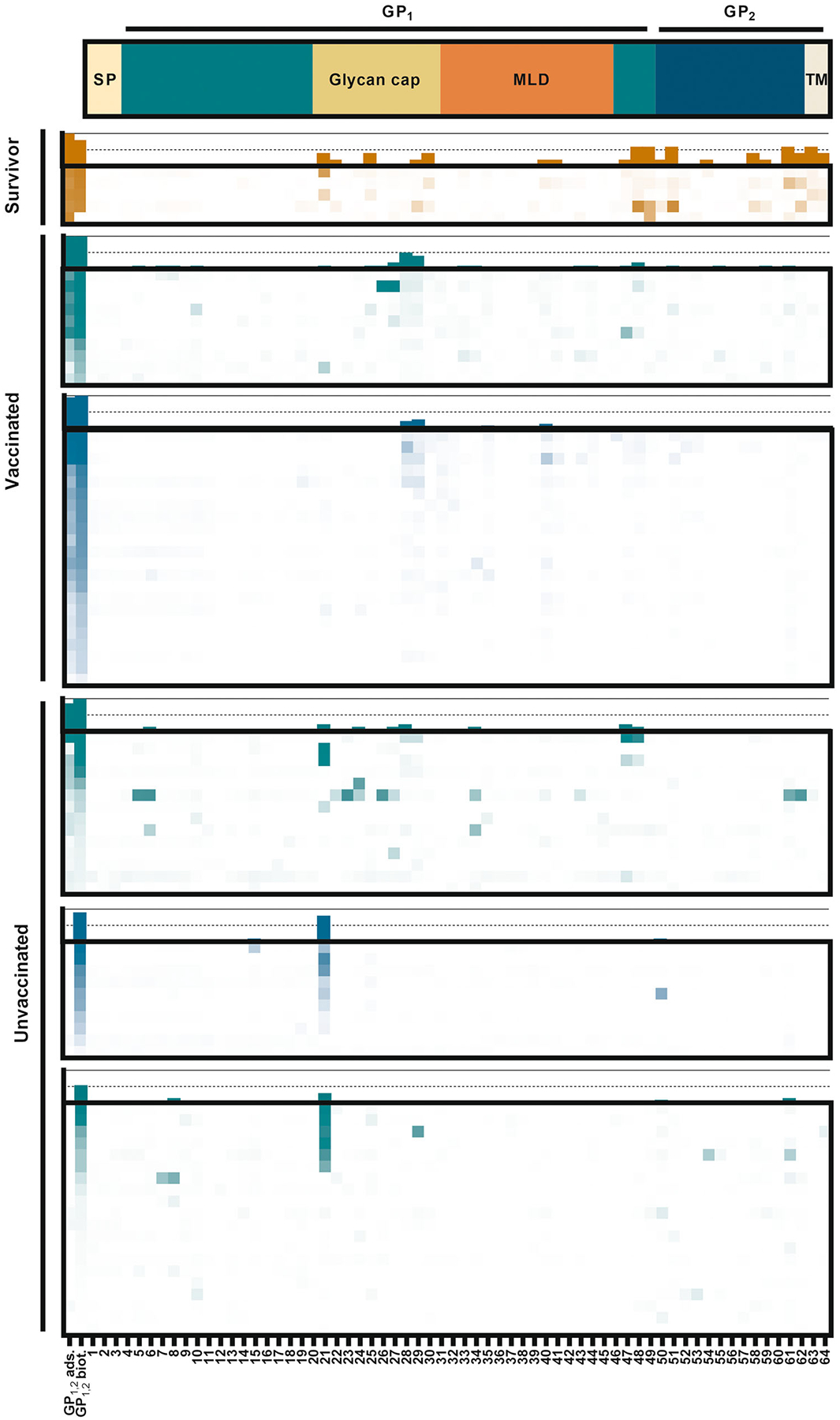
Identifying the epitope responsible for EBOV GP_1,2_ reactivity in unvaccinated, uninfected individuals. Sera from survivors, vaccinated individuals and unvaccinated individuals were tested against an array of 25-residue long, 15-residue overlapping peptides representing EBOV GP_1,2_ residues 1–655 out of 676. The schematic at the top shows the major features of the EBOV GP_1,2_ sequence. Individual donors are depicted in rows; antigen trimer and peptides, in columns. The heatmaps represent background-corrected absorbances between 0 and 4 units. Frequencies of positive signals are shown as columns above each panel with dashed line indicating 50% and solid line 100%. Ochre: samples collected in Liberia; teal: US; green: Uganda. SP, signal peptide; GP_1_ and GP_2_, glycoprotein subunit 1 and 2; MLD, mucin-like domain; TM, transmembrane domain. Donors from Uganda who were identified as unvaccinated are divided into two panels based on previously noted reactivity against biotin-captured antigen.

To identify the epitopes responsible for this phenomenon, sera from unvaccinated individuals showing increased binding with adsorbed antigen, sera with no binding as negative controls, and sera from vaccinees, survivors, and all those with reactivity against biotin-captured antigen but no reported vaccine history identified in our earlier study^24^ were subjected to an epitope-mapping scan. In this screen, the sera were tested against 25 residue-long peptides that overlapped by 15 residues and represented 97% of the length of EBOV variant Kikwit GP_1,2_ (residues 1–655 of 676, excluding the C-terminus of the transmembrane domain for which peptides could not be synthesized; Figure 2). Overall, epitope usage among survivors from Liberia and vaccinated individuals from Uganda and United States varied between donors, and some donors had no overt reactivity towards any of these linear epitopes. Lack of linear epitope recognition is not completely unexpected, since only approximately half of monoclonal antibodies isolated from vaccinees were found to bind linear peptide epitopes despite binding the complete GP_1,2_.^26^ Of the vaccinated individuals, 10 of 32 (31.3%) had reactivity towards peptides 28 and/or 29, corresponding to residues 271–295 and 281–305 within the GP_1_ glycan cap domain, respectively. Such reactivity was virtually absent in those samples from US and Uganda that only reacted when the GP_1,2_ antigen was nonspecifically bound to assay surface or did not react to GP_1,2_ at all (1/30 donors P29-reactive; Fisher’s exact test *p* = 0.0061). Three out of 14 (21.4%) Uganda healthcare worker donors recorded as unvaccinated but whose sera reacted to biotin-bound antigen in our earlier work did bind P28/29, suggesting that this group from a retrospective study (total *N* = 352,^24^) may include a handful of vaccinees misidentified as unvaccinated.

In contrast to these weak signals, moderate to strong reactivity towards a single peptide (P21; GP_1_ residues 201–225 within the glycan cap) was frequently observed among samples that bound GP_1,2_ only when nonspecifically adsorbed to assay matrix. Reactivity towards this peptide was also occasionally observed among those vaccinated or those who have survived EBOV infection. Further efforts concentrated on this epitope due to the high frequency and potent signals among those expected to be seronegative towards EBOV GP_1,2_.

**FIGURE 3:**
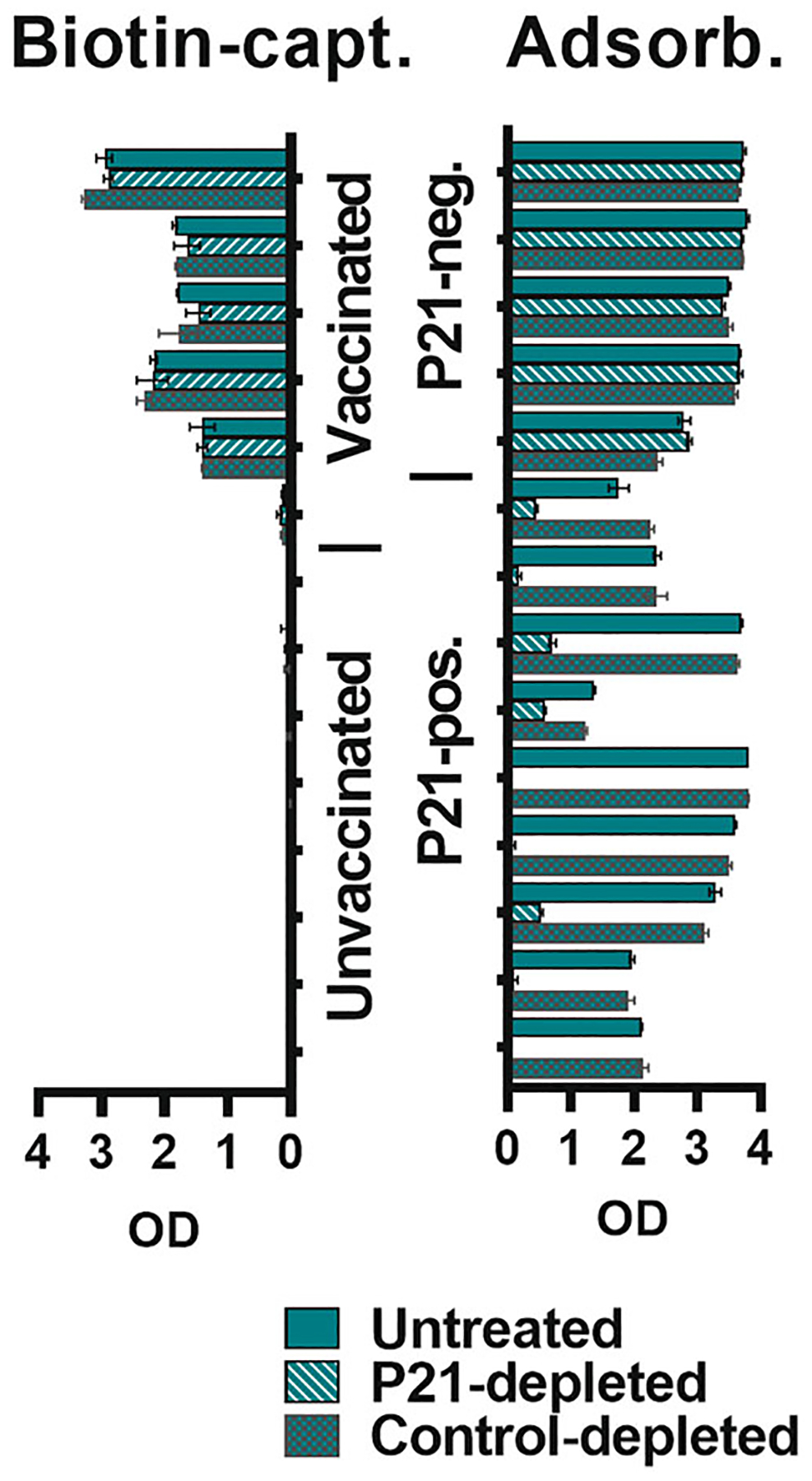
Verifying the role of P21 binding-antibodies in unspecific reactivity towards the EBOV GP_1,2_ trimer. Sera were depleted from antibodies binding peptide P21 (or a negative control peptide, P53) and tested for remaining reactivity against biotin-captured or adsorbed EBOV GP_1,2_ trimer. Six samples from vaccinees, out of which one bound P21, and eight from unvaccinated individuals, all of which bound P21, are depicted as groups of three bars each. Absorbances from a single dilution (1:200) represented with ranges of technical duplicates. OD, optical density.

Observing reactivity towards a single linear epitope does not necessarily exclude the presence of further antibodies binding conformational epitopes and contributing to reactivity towards the GP_1,2_ trimer. To assess the relative contribution of the epitope within P21, a subset of sera identified as P21-binders in Figure 2 together with sera from vaccinated controls were depleted of antibodies binding P21 (Figure 3). P21 depletion markedly reduced reactivity towards adsorbed antigen, with all tested sera identified as P21-binders, including serum from a vaccinee who had low titers against biotin-captured antigen. As expected, sera from vaccinees that did not bind P21, bound the trimer equally well after P21 depletion. These data confirmed that an epitope within the P21 sequence was critical for the apparent cross-reactivity against the complete GP_1,2_ antigen trimer.

**FIGURE 4:**
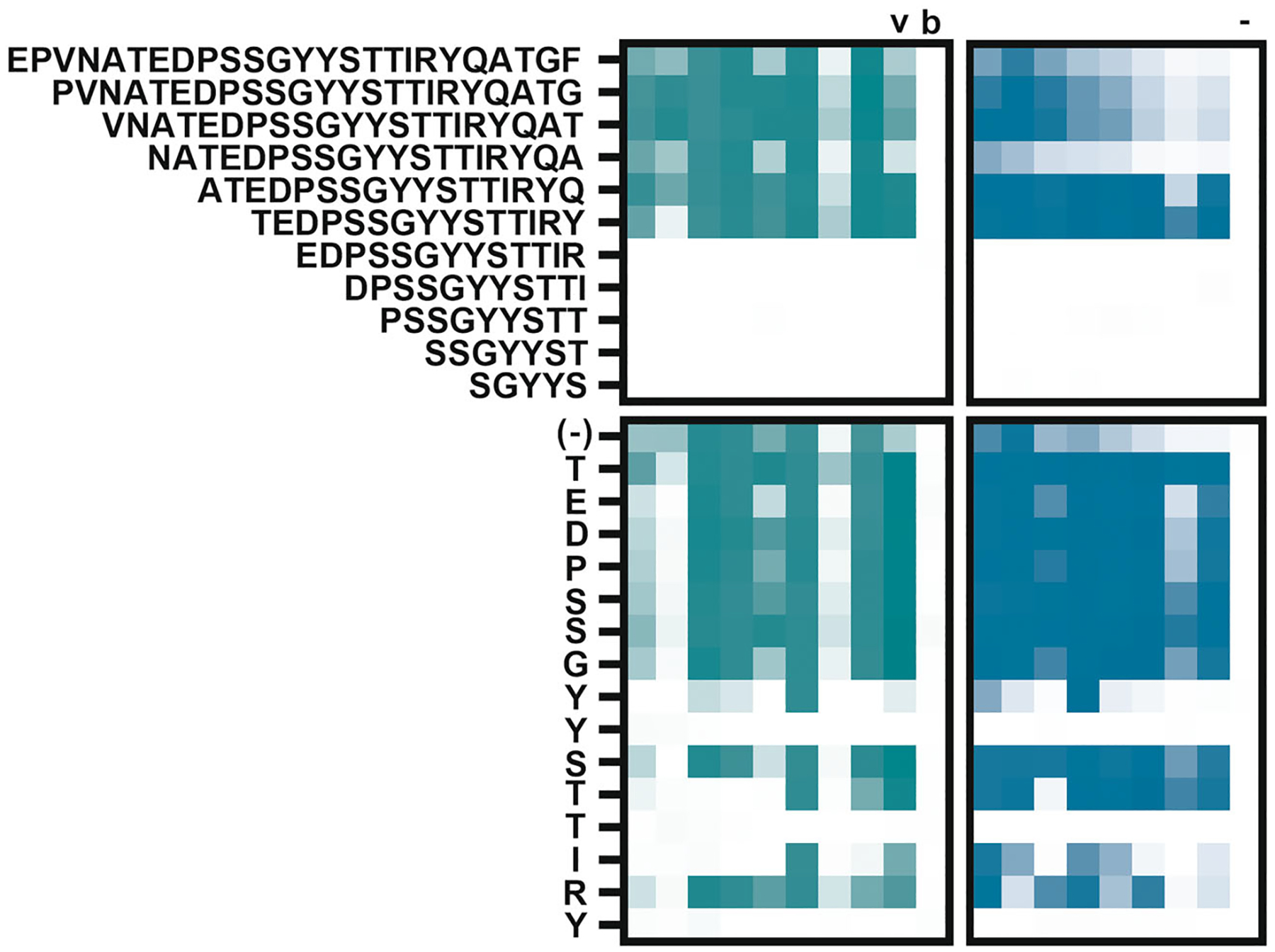
Identifying residues critical for antibody binding within the P21 sequence. Top: P21 (first row) was shortened from both ends, and P21-reactive sera (and a negative control) were tested for loss of reactivity. Bottom: Each residue of the shortest reactive peptide identified above was changed to alanyl and sera were tested for loss of reactivity to identify residues critical for antibody binding. Each column represents an individual serum sample. The colors in the heatmaps represent background-corrected optical densities between 0 and 4 absorbance units. Green: serum samples from Uganda; teal: serum samples from US. v, sample from vaccinated individual that bound P21; b, blank; -, control sample that did not bind P21.

Next, critical antibody-binding residues defining the epitope were identified in a two-step process. First, the 25-residue long sequence of P21 was shortened from both ends until P21-binding sera lost reactivity (Figure 4). Strikingly, all tested sera from both Uganda and US donors lost reactivity when the peptide was shortened from 15 to 13 residues. In the second step, those 15 residues were changed one by one to alanyls. All tested sera exhibited notably similar binding characteristics, with three residues (corresponding to EBOV GP_1_ residues Y214, T217, and Y220) critical for binding in all cases. Differences between donors were noted in the requirement for adjacent residues. Of note, many sera appeared to bind the shortened peptides better than the parental P21 peptide. This may be due to changes in secondary structure adopted by the peptides and may help to explain why no P21-binding serum was found to bind P22 although the critical residues are within the overlap of these two peptides (Figure 2). Alternatively, two or more residues within the unique part of P21 may require concurrent changes to reveal their contribution to the epitope.

**FIGURE 5:**
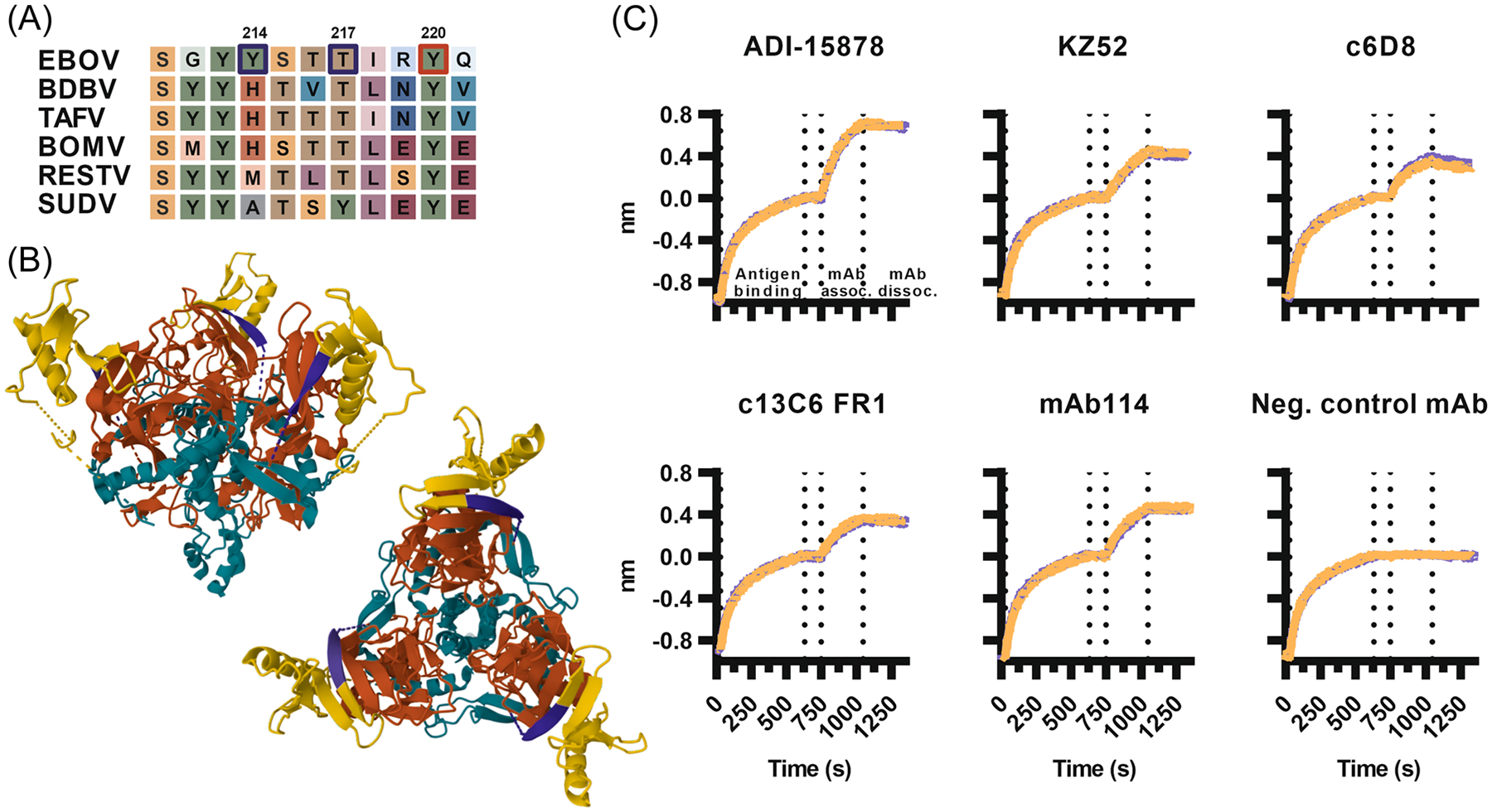
Generating a variant EBOV GP_1,2_ antigen to minimize unspecific binding. (A) Alignment of the identified epitope region in the glycoprotein sequence of EBOV (variant Kikwit; Uniprot P87666) and the other known viruses in the genus *Orthoebolavirus*: Bundibugyo virus (BDBV; Uniprot B8XCN0), Taï Forest virus (TAFV; Q66810), Bombali virus (BOMV; A0A4D5SG72), Reston virus (RESTV; Q66799), and Sudan virus (SUDV; Q7T9D9). Three residues critical for antibody binding are highlighted: two that could be changed to alanyls without impacting protein production in purple (Y214, T217), and one that could not in red (Y220). Graph created with BioRender.com. (B) Location of the epitope on the EBOV GP_1,2_ trimer. Image from the RSCB protein database (RCSB.org) of the crystal structure 6VKM (https://doi.org/10.2210/pdb6VKM/pdb^21^) highlighting solved parts of GP_1_ in red, glycan cap within it in yellow, and the epitope in purple. Side and top views. (C) Biolayer interferometry graphs depicting binding of monoclonal antibodies to wild-type antigen (orange) or variant antigen (Y214A/T217A, purple).

Identifying residues critical for the apparent cross-reactivity enabled exploration of improved antigen design. Although three residues (Y214, T217, and Y220) were each individually necessary for binding by all tested sera, triple and double changes to alanyls were attempted for redundancy. Out of the 3 possible double changes and the triple change, only the Y214A/T217A variant trimer could be expressed at levels similar to those of wild-type antigen and adequate for purification (Figure S1). This indicated that Y220, the only identified residue absolutely conserved among the orthoebolaviruses (Figure 5A), could not be changed to alanyl without compromising the yield. In the trimer structure (Figure 5B), Y214 is located within an unmodeled stretch, while T217 and Y220 lie within a sheet in the glycan cap domain.

As the first step to assess the impact of the residue changes on folding and antigenicity, binding of monoclonal antibodies to wild-type and variant antigens was compared by biolayer interferometry. mAbs binding GP_1_ and GP_2_ at the base (ADI-15878^22,27^, KZ52^28,29^), a linear epitope in the mucin-like domain of GP_1_ (c6D8,^30,31^), GP_1_ glycan cap (c13C6 FR1^30–32^), or glycan cap and GP_1_ core (mAb114^23,33^) retained wild-type-like binding of the Y214A/T217A variant trimer, while a negative control antibody bound neither (Figure 5C). Comparable mAb binding with the wild-type trimer suggested that the variant antigen retained overall fold and accessibility of tested epitopes.

**FIGURE 6:**
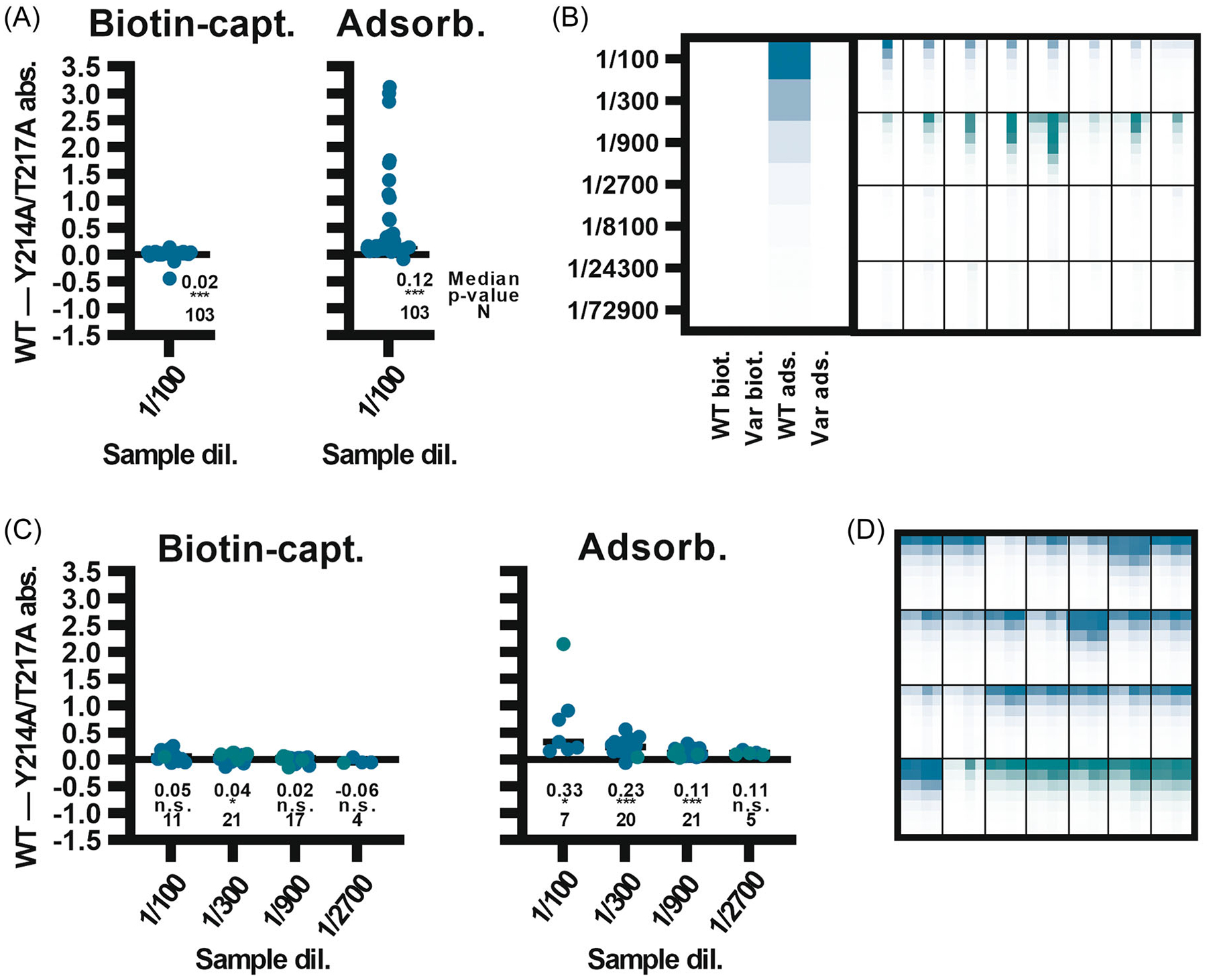
Impact of Y214A/T217A changes on the performance of trimeric EBOV GP_1,2_ antigen. Serum samples identified as binders of the cross-reactive epitope, as non-binders of the cross-reactive epitope, or from vaccinated individuals were analyzed using the wild-type (WT) or the Y214A/T217A (Var) antigen. (A) Unvaccinated individuals and the absolute difference in background-corrected absorbances when comparing the WT and Var antigens in either biotin-captured or adsorption formats. (B) Heatmaps including a subset of samples from (A) illustrating the signal differences at various dilutions (background-corrected absorbances between 0 and 4). One panel enlarged. (C) Difference in WT and Var antigen-corrected absorbances in vaccinee samples. Differences were calculated for samples and dilutions for which both replicates of 2 repeated experiments fell within meaningful quantitation range of 0.35–3 absorbance units. (D) Heatmaps of samples from vaccinated individuals (those in C). In (A) and (C), difference to no-difference hypotheses were analyzed using one sample Wilcoxon signed-rank test. N.s., not significant; *, *p* < 0.05; **, *p* < 0.01; ***, *p* < 0.0001. Samples from US in teal, and from Uganda in green.

As the second step, samples from unvaccinated and vaccinated donors were assayed against wild-type and variant antigens in adsorbed and biotin-captured formats. In the biotin-captured format, minimal differences in signal were observed between the antigens, although statistical significance was seen with negative donor samples (Figure 6A, C). These data verified that the identified epitope is irrelevant for measuring vaccine responses in the context of a stabilized antigen captured by its C-terminus, corresponding to the transmembrane domain.

In contrast, when the antigens were adsorbed directly to the assay matrix, a marked reduction in nonspecific signals was observed when using the variant antigen (Figure 6B). Apart from the minority of samples from unvaccinated individuals with high titers against the wild-type antigen, the median signal from 103 US donors was also modestly elevated against the wild-type trimer (Figure 6A). These wild-type signals correlated well with those measured using another EBOV GP_1,2_ antigen^34^ and the correlation was diminished when using the variant antigen (Figure S2), implying that conclusions from this work are applicable beyond the in-house antigen used in the experiments. Importantly, nonspecific signals in samples from unvaccinated donors were occasionally strong (up to endpoint 1/8100), rivaling true signals from vaccinees (Figure 6B, D). This demonstrates that qualitative changes in the antigen, instead of mere optimization of conditions and cut-offs, were required to improve performance of EBOV GP_1,2_ serology. As expected from the results produced with samples from negative donors, adsorbed wild-type antigen also resulted in modestly elevated signals as compared to the variant antigen when samples from vaccinated donors were tested (Figure 6C, D).

## 4 | DISCUSSION

This work identifies a single epitope largely responsible for the often-observed reactivity amongst individuals expected to be antibody-negative against EBOV GP_1,2_. We conclude that this signal represents cross-reactivity with an antigen unrelated to EBOV GP_1,2_, since (1) antibodies of donors from Africa, where orthoebolaviruses are known to circulate, and from Northern America, where they are not, were found to bind the same epitope; (2) binding to the full antigen appeared to be completely or nearly completely mediated by this single epitope; and (3) binding was not commonly observed among those vaccinated against EBOV GP_1,2_. Importantly, all tested sera required a core set of antigen residues, pinpointing residue changes that could improve the antigen. The source of the cross-reactivity itself remains unknown. The epitope sequence is short with only a few residues found critical for antibody binding. NCBI-BLAST protein database searches with the sequence limited by critical residues yielded diverse hits, none of which was considered a clear candidate for further experimentation. Indeed, the source of the observed cross-reactivity may be part of a vaccine, pathogen, the environment, or even self.

Identification of a single epitope (and indeed single amino acid residues) that can be modified to decrease background and improve EBOV assay performance across individuals seems serendipitous. However, despite the immense theoretical diversity of antibody sequences, recognition of the same viral peptides by sera collected around the world may in fact be commonplace.^35^ Mechanistically, epitopes may become “public” this way when germline antibody sequences are important for affinity, while divergent “private” epitopes would arise from greater contribution by the most variable parts of an antibody.^36^ Therefore, it seems plausible that public epitopes and antibodies cause false positivity against other antigens as well, and that workflows such as the one here can be used for broad improvements in the field of serology.

In the presented case of EBOV GP_1,2_, the cross-reactive epitope appears to be only exposed under standard antigen-coating conditions, and assay specificity was similarly improved by coating via C-terminal biotin capture. Of note, another assay in which GP_1,2_ binding to assay surface is circumvented (here, by capturing serum antibodies and detecting them with labeled antigen)^37^ achieved complete specificity among 339 controls while retaining high sensitivity.^38^ Such performance is a high bar to reach. Indeed, the expansion of negative control numbers here identified a single US donor with low reactivity above the pre-determined cut-off in the assay format using wild-type GP_1,2_ trimer with biotin-mediated coating (one positive out of combined 195 US negative donors^24^). Other approaches to increase confidence in EBOV serology results include verification testing against the same antigen using different platforms^39^ and testing against two or three different viral proteins (which is only applicable for identifying infected individuals rather than those vaccinated against a single viral protein), although rare multi-antigen reactivity is still seen in a population expected to be seronegative.^19^

In addition to characterizing the cross-reactive sequence, our peptide screen also helps to identify GP_1,2_ epitopes targeted by the human antibody response. Regarding vaccinees, this screen identified a single glycan cap region targeted by a considerable minority of vaccinees from both US and Uganda. This may indicate that the antibody response largely targets conformational epitopes that cannot be identified with peptides, although the glycan cap itself is a frequent target of both human and mouse antibodies.^40^ Previous studies have mapped vaccinee and survivor serum reactivity against GP_1,2_ epitopes using ELISA^41^ peptide chips^42,43^ and phage-display libraries.^44,45^ All in all, small survivor sample sets both here and in other published works are a caveat that limits conclusions when it comes to comparing B-cell epitopes recognized by vaccinees and survivors. Authors of the phage-display work considered reactivity to be greater towards regions near C-terminal parts of GP_1_ and GP_2_ in a survivor^45^ than in pooled samples from prime-boost recipients of the rVSV-ΔG-EBOV-GP vaccine,^44^ and our results with single-dose vaccinees appear to be in line with that interpretation. Peptide chips also identified at least partially overlapping epitopes in the same regions,^43^ while others found that survivor reactivity targeted linear epitopes in GP_1_.^41^ The observation that shortening a peptide (or making specific changes to it) can increase signal implies that the choice of peptide length and other technical variables can impact results from epitope mapping experiments. In aggregate, the available data suggest that the polyclonal response against GP_1,2_ may not be identical in rVSV-ΔG-EBOV-GP vaccine recipients and survivors, although at the monoclonal level, survivor-like antibodies are found in vaccinees.^26^

In summary, the findings presented here point to a mundane explanation in case of many individuals who, contrary to expectation, test positive for antibodies against EBOV GP_1,2_. The results underscore the need for caution when interpreting rare positive findings in serology. On the other hand, new orthoebolaviruses have been discovered twice in the last two decades, once when investigating an outbreak of human disease (in the case of BDBV^46^) and once when screening animals for the presence of viral nucleic acids (in the case of BOMV^47^). Furthermore, another one (TAFV) has only emerged once, almost 30 years ago, and has not been detected since.^48^ The time of discovery may not be over when it comes to pathogens related to EBOV.

## Supplementary Material

FigureS1

FigureS2

## Data Availability

Data available within the article and its supplementary materials.
