# Supplementary figures and images for: A public, cross-reactive glycoprotein epitope confounds Ebola virus serology
Source: J Med Virol. Author manuscript; Available in PMC 2025 Mar 3. (PMC11874798; doi:10.1002/jmv.29946)

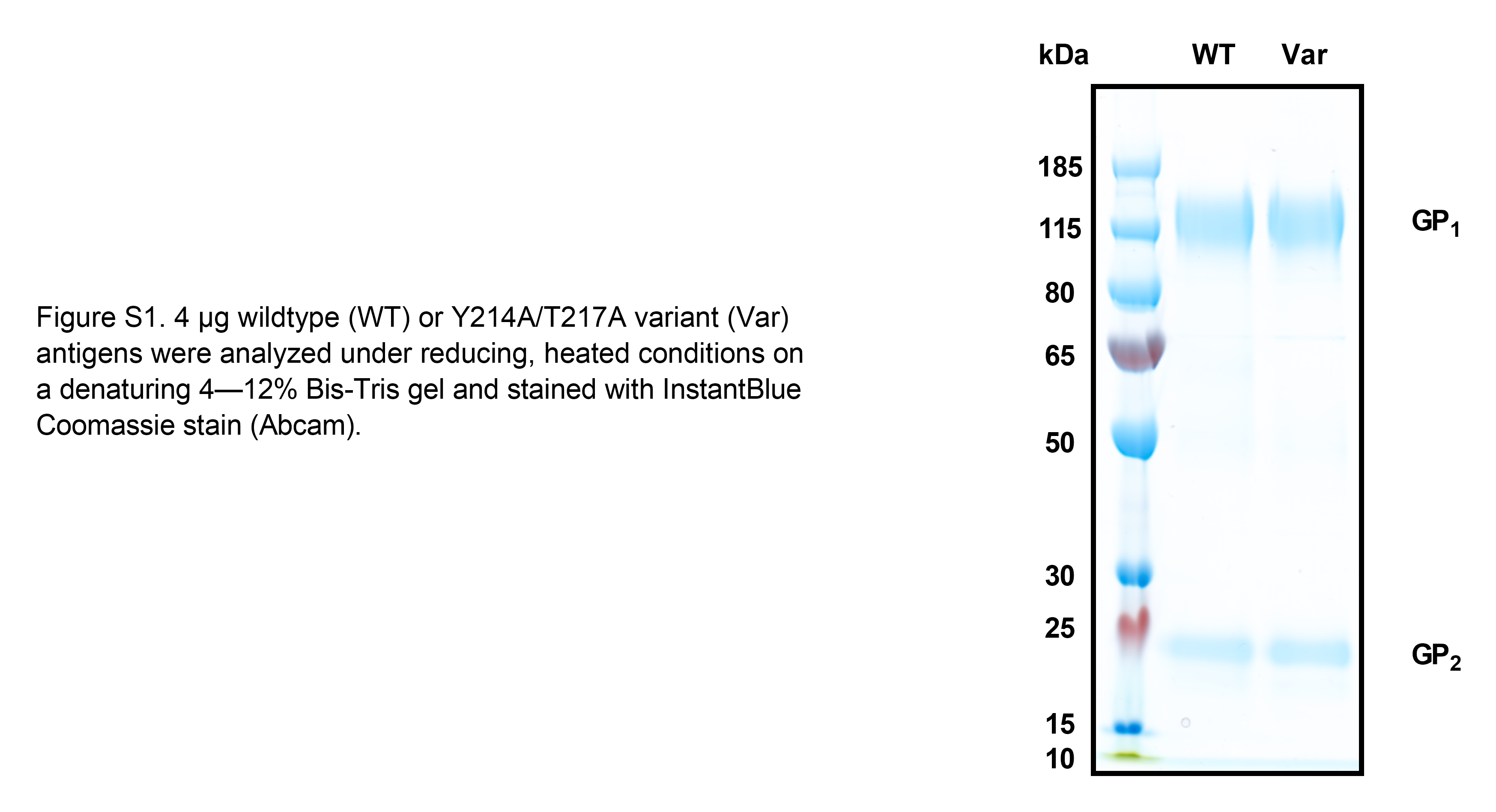

Supplement: FigureS1 [file NIHMS2059591-supplement-FigureS1.tif]

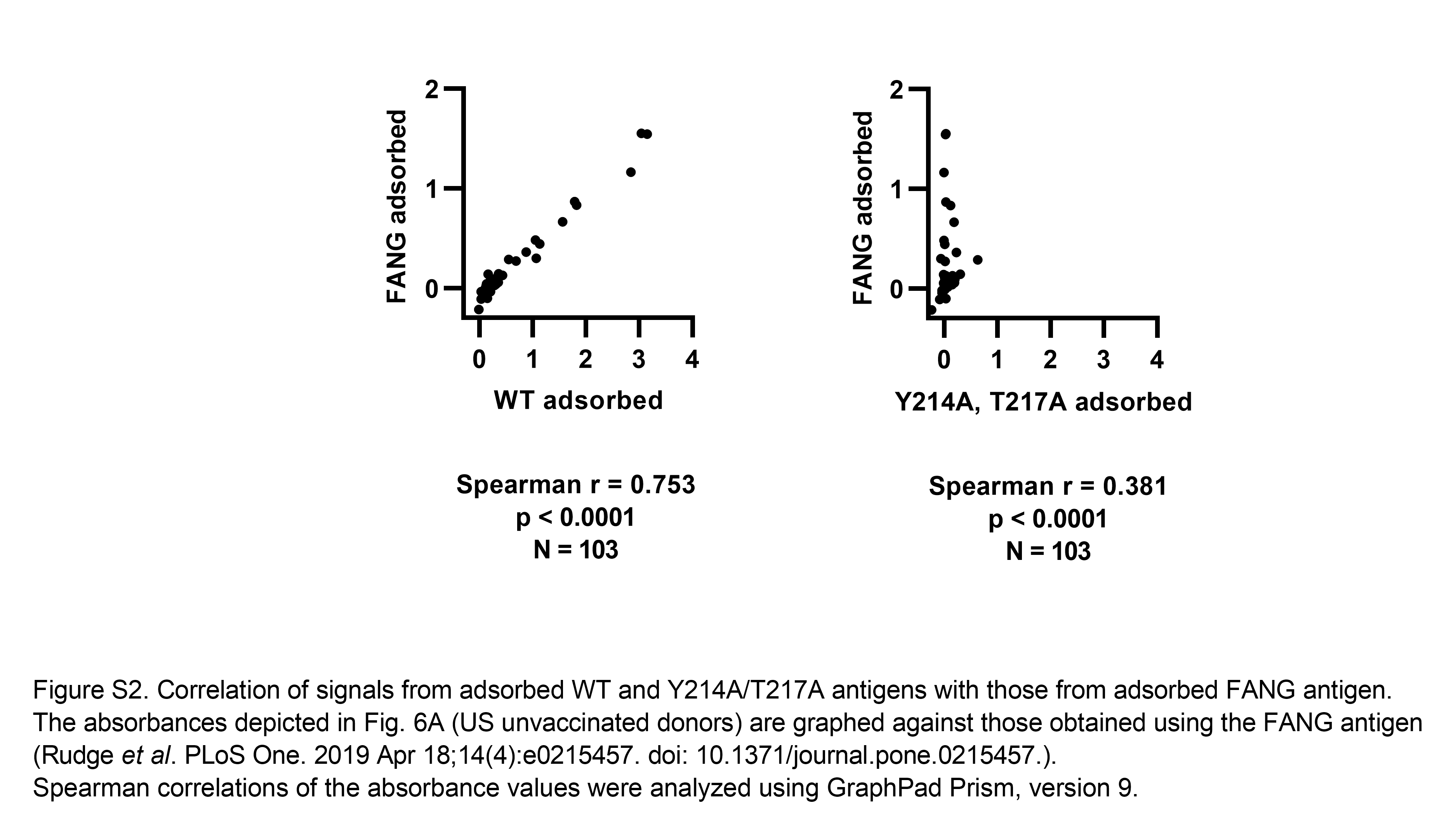

Supplement: FigureS2 [file NIHMS2059591-supplement-FigureS2.tif]
